# Identification of Seven in absentia homolog 2 as a potential efferocytosis-related biomarker in diabetic foot ulcers

**DOI:** 10.1371/journal.pone.0334163

**Published:** 2025-11-03

**Authors:** Jiangli Zhao, Xuchen Liu, Qingyuan Sun, Yanya He, Jiwei Wang, Junzhi Liu, Chao Li, Fei Yan, Haoyong Jin, Zhiwei Xue, Ziyi Tang, Nan Su, Ning Yang, Xinyu Wang

**Affiliations:** 1 Department of Neurosurgery, Qilu Hospital, Cheeloo College of Medicine and Institute of Brain and Brain-Inspired Science, Shandong University, Jinan, China; 2 School of Medicine, Cheeloo College of Medicine, Shandong University, Jinan, China; 3 Jinan Microecological Biomedicine Shandong Laboratory, Jinan, China and Shandong Key Laboratory of Brain Function Remodeling, Jinan, China; 4 Department of Endocrinology, Qilu Hospital, Cheeloo College of Medicine, Shandong University, Jinan, China; 5 Jinan Clinical Research Center for Endocrine and Metabolic Diseases, Jinan, China; Gachon University Gil Medical Center, KOREA, REPUBLIC OF

## Abstract

**Introduction:**

Wound of diabetic foot ulcers (DFU) is chronic and hard to heal, characterized by impaired inflammatory response, dysfunction of keratinocyte and endothelial cells and improper removal of dying cells. Efferocytosis, as a trigger for phenotype switch of macrophages, plays a critical role in diabetic foot wound healing. Here, we showed the effect of efferocytosis in wound healing of diabetics and identified seven in absentia homolog 2 (SIAH2) as a potential efferocytosis-related biomarker.

**Methods:**

Blood and skin samples were collected from 20 patients diagnosed type II diabetes at Qilu Hospital of Shandong University. Efferocytosis related genes in DFU were identified based on GSE147890, GSE80178 datasets as well as RNA-seq data of blood samples. Enrichment analysis, clustering analysis and protein-protein interaction network analysis were conducted based on the efferocytosis related genes in DFU. An array diagram was constructed and survival analysis of DFU was performed based on the associated clinical data. Single-cell sequencing data analysis combined with experiments in vitro, we analyzed the role of SIAH2 in wound healing of DFU as well as its correlation with efferocytosis signal.

**Results:**

Overall efferocytosis and SIAH2 expression level were increased in DFU blood and tissue samples and associated with poor survival in patients. Single-cell analysis revealed elevated SIAH2 expression is positively associated with keratinocyte migration, angiogenesis and efferocytosis of macrophage in wound healing of DFU. SIAH2 involved in efferocytosis-related cell-to-cell communication, especially in “internalization” and “digestion” signals.

**Conclusion:**

SIAH2 was identified to be one of the key efferocytosis genes and associated with poor prognosis of DFU. Protective upregulation of SIAH2 was involved in angiogenesis, keratinocyte migration and cell-to-cell communication mediated by efferocytosis in DFU wound healing.

## Introduction

Diabetic foot ulcer (DFU) represents a debilitating diabetes-associated complication characterized by elevated morbidity and disability risks [1]. The sensory disturbances, pain, and motor function impairment associated with DFU severely impact the quality of life of patients [2,3], while the accompanying infections often lead to gangrene and subsequent amputation [4,5]. Although effective training can be employed to improve self-care awareness and help prevent DFU [6], the treatment primarily revolves around routine debridement and anti-infection measures. Additional interventions, such as wound aspiration and electrical stimulation, can improve clinical symptoms and prognosis in certain cases [7,8]. However, most symptoms are irreversible, and the molecular mechanisms underlying diabetic foot ulcer treatment require extensive research.

Efferocytosis is a crucial process by which phagocytes efficiently eliminate apoptotic cells and maintain tissue homeostasis [9]. Downstream molecular pathways are activated through efferocytosis, including anti-inflammatory pathways, which clear cells and release cytokines to promote cell renewal [10,11]. Defects or mutations in the efferocytosis mechanism have been associated with various human diseases, including atherosclerosis, cancer, and diabetes [12–14]. Recent studies have highlighted the important role of efferocytosis-related gene families, such as SLC7A11, in the development of DFU [15,16]. However, the regulatory network of efferocytosis in DFU is complex, and numerous efferocytosis-related genes remain unidentified. Unfortunately, there is currently no comprehensive research to identify the comprehensive expression of efferocytosis related genes in DFU patients. The specific regulation of factors such as skin healing and angiogenesis during the progression of DFU disease by efferocytosis is still unclear.

In this study, we integrated skin tissue data from the Gene Expression Omnibus (GEO) dataset and compared them to the transcriptome sequencing results obtained from blood samples collected from patients at Qilu Hospital. Efferocytosis level of the specific checkpoints in the sequencing data were comprehensively analyzed during recruitment, recognition, internalization, and digestion. The sequencing results indicated a series of differentially expressed genes related to efferocytosis, among which SIAH2 (Seven in absentia homolog 2), an RING E3 ubiquitin ligases, is particularly noteworthy. SIAH2 was known as the regulation role of the SIAH2-HIF-1 axis which promoted the adaptation of cells to oxygen deprivation (hypoxia) [17]. James N Musyoka’s studies suggested that SIAH2 plays a crucial role in skin wound repair [18]. SIAH2 is known to regulate the immune system and control anti-tumor immunity [19,20] and is also believed to promote the production of neovascularization and may affect keratinocytes’ migrating through partial Epithelial-Mesenchymal Transition. But the corresponding in vitro and in vivo validation is not yet sufficient. However, there is not yet sufficient evidence to suggest that SIAH2 may play a role in the progression of DFU. Our research preliminarily confirmed the expression of SIAH2 is upregulated in DFU patients, but it is unclear whether it is related to prognosis.

In this study, whether SIAH2 was an efferocytosis-related differentially expressed gene that plays a critical role in the progression of DFU was identified. And we found patients with lower SIAH2 expression exhibit poorer clinical prognosis, including increased risk of amputation, and slower wound healing rates than those with higher expression. These observations were attributed to impaired keratinocyte function and neovascularization at the ulcer site and compromised cell clearance, likely resulting from dysfunctional efferocytosis. Further research on the role of SIAH2 in DFU is warranted because it holds promise for molecular pathogenesis analysis, diagnostic criteria, and therapeutic targets.

## Methods

### Ethics statement

This study was approved by the Medical Ethics Committee of Qilu Hospital of Shandong University (Approval No. KYLL-20210701) and was conducted in accordance with the principles of the Declaration of Helsinki. Written informed consent was obtained from all individual participants. The approval document authorizes the collection of blood samples and intraoperative skin tissue specimens from consenting DFU patients at Qilu Hospital from June 1, 2022 to July 20, 2023.

### Study participants and clinical samples

RNA expression data from blood samples were collected from patients at Qilu Hospital in Jinan, China. The inclusion criteria for patients with DFU were as follows: (i) diabetes mellitus duration of > 3 years; (ii) presence of neuropathy; (iii) age > 21 years; and (iv) foot wound duration > 4 weeks. Patients were excluded if they had: (i) active cellulitis; (ii) osteomyelitis; (iii) severe septicemia; (iv) had undergone revascularization to the ipsilateral lower extremity within the past 6 weeks; and (v) had used any experimental drugs orally, by injection, or topically within the 4 weeks preceding the study. RNA expression data were obtained using the microarray assay test, and detailed methods and clinical information can be found in S2 Table. Whole blood samples were cryopreserved at −80°C until RNA extraction. RNA expression data were collected from the whole blood of 10 patients with DFU, while data from ten individuals with diabetes mellitus served as the control group. The skin samples from DFU patients were obtained from debridement and amputation operations to treat DFU, while the normal skin samples were taken from amputation surgeries to treat lower limb injuries caused by car accidents. The collection of tissues and blood commenced on June 1, 2022, and concluded on July 20, 2023.

### GEO data downloading and processing

The datasets from Gene Expression Omnibus (GEO) database were selected according to the following criteria: (1) RNA-seq or microarray data; (2) inclusion of diabetic foot/unhealed diabetic foot ulcer and non-DF skin tissue samples; (3) no prior artificial intervention or medication; and (4) human origin. Based on these criteria, a search of the GEO database using the term “diabetic foot” identified three datasets that met all inclusion requirements (GSE80178, GSE68183, and GSE134431) [21]. The R package “sva” was used to combine the datasets and remove inter-batch differences [22]. Ultimately, a dataset combined by GSE80178 and GSE68183 consisting of 17 samples, including six non-Diabetic Foot (non-DF) Skin, six diabetic foot skin (DFS), and five DFU samples, was obtained. For validation, we used diabetic foot skin and non-healing diabetic foot ulcer samples from GSE134431, excluding healed diabetic foot ulcer cases to minimize potential confounding factors. In addition, single-cell RNA sequencing (scRNA-seq) data from dataset GSE165816 were used for data processing and analysis of the foot skin of patients with and without DFU.

### Acquisition of human protein atlas data

Images of immunohistochemical sections and the molecular structure of SIAH2 were obtained from the Human Protein Atlas (HPA) (https://www.proteinatlas.org/). The HPA provides comprehensive lists of proteins expressed at elevated levels in different tissues to provide a spatial context, including the localization of proteins in sub-compartments of each tissue and organ at the single-cell level [23].

### Obtaining efferocytosis-related genes

Efferocytosis-related genes (ERGs) were obtained from the GeneCards database (https://www.genecards.org/), which is a portal website and database containing information on more than 155,000 human genes, including their functions, protein domains, and interactions [24]. A search for “efferocytosis” in GeneCards was performed to obtain ERGs. Genes that were unrelated to efferocytosis or lacking protein products were removed, resulting in 100 acquired genes. A full list of ERGs is provided in S1 Table.

### Identification of differentially expressed ERGs

Differential analysis was performed using the “limma” package in R (version 3.56.2), an algorithm that integrates data from gene expression experiments [25]. The cut-off criteria used for filtering differentially expressed genes (DEGs) were set as “|log2FC| > 1 and adjusted P value < 0.05,” unless otherwise specified.

### Conduction of single sample gene set enrichment analysis

The ssGSEA scores were calculated using the “GSVA” package in R (version 1.48.3) software. GSVA provides increased power to detect subtle pathway activity changes in a sample population compared to other methods. ssGSEA was used to estimate the activity of cell death pathways, efferocytosis, immune functions, and the relative abundance of infiltrating immune cells.

### Consensus clustering of patients with DFU

To identify distinct efferocytosis patterns based on ERGs, we employed consensus clustering using the “ConsensusClusterPlus” package in R (1.64.0) software. This method involves resampling to identify each number and subgroup number, which is then verified based on clustering rationality [26].

### Calculation of the cluster score of patients with DFU

Principal component analysis (PCA) was used to display the effect of clustering, a mathematical algorithm that reduces the dimensionality of the data while retaining most of the variation in the dataset [27]. This allowed us to calculate the cluster score of patients with DFU using the following formula: cluster score = ∑PCi.

### Functional and pathway enrichment analysis

The R package “clusterProfiler” (version 4.8.3) was used to perform Gene Ontology (GO) and Kyoto Encyclopedia of Genes and Genomes (KEGG) enrichment analyses to explore the functions and pathways associated with efferocytosis [28]. Additionally, Gene Set Enrichment Analysis (GSEA) was conducted using GSEA software (v4.2.3) to analyze the differentially expressed pathways between the two groups. Enrichment scores (ES) were calculated based on weighted Kolmogorov-Smirnov-like statistics, with higher ES values indicating a higher likelihood of enrichment in a particular group [29]. Furthermore, the function of key ERGs and their associated genes were annotated using the online tool “GeneMANIA” (http://genemania.org/), which aids in generating hypotheses about gene function, analyzing gene lists, and prioritizing genes for functional assays [30].

### Estimation of immune cell infiltration in patients with DFU

Multiple algorithms, including ssGSEA, MCPCOUNTER, TIMER, and CIBERSORT, were used to calculate the relative abundance of immune cells in blood and skin tissue. ssGSEA was conducted using the “GSVA” package in R (version 1.48.3), whereas the other three analyses were performed using the TIMER website (http://timer.cistrome.org/) to provide supplementary validation of the results. TIMER is a comprehensive website for analyzing immune cell infiltration [31].

### Construction of the nomogram model

A nomogram model was constructed using the R package “rms,” (version 6.7.1) and a calibration model was plotted to evaluate the consistency between the predicted values and the actual outcomes [32]. Additionally, decision curve analysis (DCA) and clinical impact analysis were performed to assess the potential benefits of decisions based on the model for patients with diabetic foot ulcers.

### Single cell RNA sequencing analysis

Analysis of scRNA-seq data was conducted using the “Seurat” package in R software (version 4.4.0). Quality control measures were applied, including the inclusion of genes expressed in at least three single cells, the exclusion of cells expressing 50 genes, and the removal of cells with > 10% mitochondrial genes. Normalization of the data was achieved using the “NormalizeData” function with the “LogNormalize” method, followed by transformation into a Seurat object. PCA reduction was performed based on the top 1500 highly variable genes, and the first 80 principal components were selected for UMAP reduction with a resolution of 0.5. Pseudotime analysis was conducted using the “Monocle” package in R (version 2.28.0). In addition, intercellular communication was analyzed using the R package “CellChat” (version 1.6.1). Representative markers included: DCD for sweat/sebaceous gland cells; TPSAB1 for mast cells; IGHG1 and CD79A for plasma and B cells; MLANA for melanocytes; CCL21 for lymphatic endothelial cells; EPCAM, KRT5, and KRT1 for epithelial and keratinocyte subtypes; LUM and COL1A1 for fibroblasts; CD163 and CD68 for monocytes/macrophages; CCL5 and CD3D for T/NK cells; ACKR1 for vascular endothelial cells; and ACTA2 and TAGLN for smooth muscle cells (SMCs).

### Cell culture and transient transfection

HaCaT cells, HUVECs, and Jurkat cells were obtained from the Culture Collection of the Chinese Academy of Sciences (Shanghai, China). HUVECs and HaCaT cells were cultured in Dulbecco’s modified Eagle’s medium (DMEM; Life Technologies, USA) supplemented with 10% fetal bovine serum (FBS; VivaCell, Shanghai, China). Jurkat cells were cultured in RPMI Medium 1640 (Thermo Fisher Scientific). Transient transfection of siRNA was performed using Lipofectamine 2000 (Thermo Fisher Scientific). The sequences of the siRNAs are provided in S3 Table

### Quantitative real-time PCR

Total RNA was extracted from cells using a kit from Yishan Bio, China, and cDNA was synthesized using the reverse transcription qPCR RT Kit from TOYOBO, Japan. RNA from blood and skin samples was extracted using TRIzol. qRT–PCR was performed using SYBR Green Master Mix (Roche) on the 480II Real-Time PCR Detection System (Roche). β-actin mRNA expression was used as a standard, and the results represent at least three independent experiments.

### Western blotting

Protein samples from HaCaT and HUVECs cells were lysed using RIPA buffer (P0013B, Beyotime) and quantified. After mixing with SDS sample buffer, the samples were boiled for 5 minutes. Equal quantities of protein (20 µg) were separated by SDS-PAGE and subsequently transferred to either a nitrocellulose or PVDF membrane at 4°C for 1–2 hours. The membranes were then blocked with 5% non-fat dry milk in TBST for one hour and incubated with primary antibodies overnight at 4°C. Following primary antibody incubation, the membranes were probed with HRP-conjugated secondary antibodies for one hour at room temperature. After washing with TBST, the blots were treated with ECL detection reagent and visualized using an imaging system. The following antibodies were used to incubate membranes: SIAH2 antibody (Immunoway, YT4297) and β-Tubulin (D3U1W) antibody (Cell Signaling Technology, 86298).

### Wound-healing assay

HUVECs and HaCaT cells transfected with si-NC and si-SIAH2 were seeded into 6-well plates and scratched with a pipette tip three times per well to create a consistent wound area. The cells were then incubated in a serum-free medium for 0–48 h, and images were captured every 24 h using an optical microscope. The healing distance was quantified using ImageJ software.

### Transwell assay

Transwell chambers (24 wells, Corning Inc., NY) were used for the assay. HUVECs and HaCaT cells (3*10^4^) were added to the upper chamber, and assay medium (600 μL medium containing 30% FBS) was added to the lower chamber. After incubation at 37°C for 24 h and 48 h, cells were fixed and stained with crystal violet for 20 min. Images were obtained from three random fields in each well.

### Immunofluorescence analysis

Skin tissue sections from the DFU and control group patients were fixed in a 4% paraformaldehyde solution for 15 min, followed by incubation with 1% bovine serum albumin (BSA) for 60 min. All sections were then incubated with SIAH2 antibody (Immunoway YT4297, 1:200) overnight at 4°C. Subsequently, the sections were incubated with a secondary antibody (anti-rabbit IgG, Alexa Fluor 488 Conjugate, CST#4412) for 50 min, and the nuclei were counterstained with DAPI. Confocal microscopy was used to evaluate the expression levels of SIAH2 in the co-cultures (Leica TCS Sp8).

### THP-1 culture and differentiation

A THP-1 human acute monocytic leukemia cell line was obtained from the American Type Culture Collection and grown in THP-1 culture medium containing RPMI-1640 supplemented with 10%FBS. Macrophages were differentiated from THP-1 cells in the above culture media and supplemented with 100 nM of phorbol 12-myristate 13-acetate (PMA) for 72 h at a seeding density of 1*10^6^ cells per well in a 6-well tissue culture plate. PMA-containing medium was then removed and replaced with THP-1 culture medium for 48 h of culture.

### Induction and fluorescent labeling of apoptotic cells

Apoptotic Jurkat cells were created by treating Jurkat cells with 1 μM staurosporine for 4 h at a density of 2.5*10^6^ cells/ml at 37°C and 5% CO2, after which they were resuspended at a concentration of 2*10^7^ cells/mL in Diluent C with PKH26 (red fluorescence) per the manufacturer’s instruction and PBS. After labeling, the cells were rinsed twice with RPMI-1640 medium containing 10% HI-FBS and cultured further.

### In vitro efferocytosis and phagocytosis assays

Macrophages were plated in 6-well plates at a density of 3*10^5^ macrophages per well. For flow cytometry-based quantification, macrophages were plated in 6-well plates at a density of 1.5*10^6^ macrophages per well. Fluorescently-labeled apoptotic cells were co-incubated with macrophages at a ratio of 5 apoptotic cells to 1 macrophage for 2 h at 37°C and 5% CO2. The macrophages were then washed with 1X PBS gently to remove unbound targets. They were then digested with trypsin for 20 minutes and resuspended with 1 ml PBS, after which their grouping situation was analyzed using flow cytometry.

### Statistical analysis

Statistical analysis was performed using R software (version 4.31) and GraphPad Prism (version 8.3.0). Spearman linear regression analysis was used to calculate correlations between two factors. A significance level of P < 0.05 was considered statistically significant unless otherwise stated.

## Results

### Overview of efferocytosis in diabetic foot ulcer compared to diabetes mellitus

**Fig 1:**
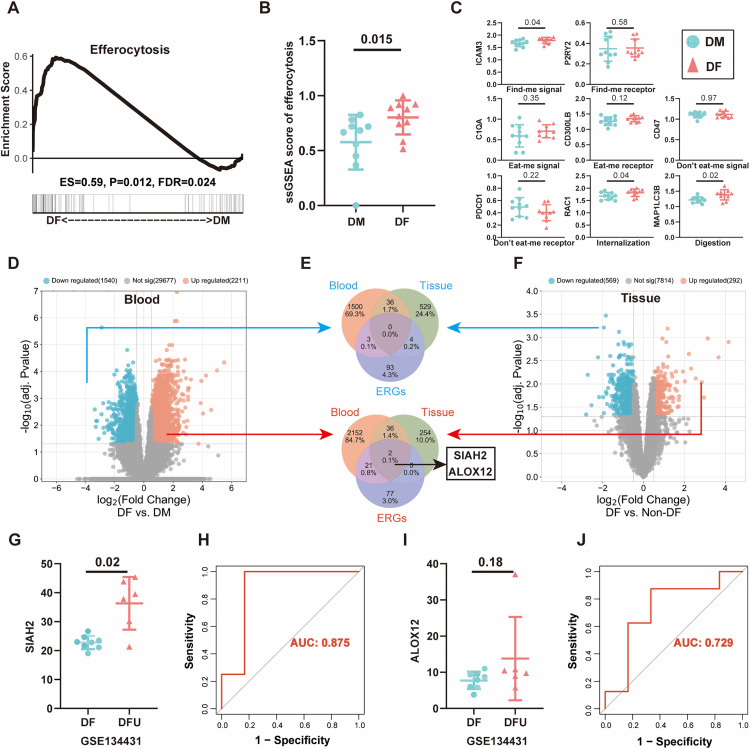
Overview of efferocytosis in DFU and identification of key ERGs. (A) GSEA of efferocytosis between DFU and DM, demonstrates that the efferocytosis gene set is significantly enriched in DF relative to DM (ES = 0.59, P = 0.012, FDR = 0.024);(B) Differential analysis of ssGSEA scores for efferocytosis between DM group and DF group (P = 0.015) (C) Differential analysis of key markers of “Find-me signal” (ICAM3, P2RY2),”Eat-me signal” (C1QA, CD300LB), “Don’t eat me signal (CD47), “Don’t eat me signal receptor (CD47), Internalization (RAC1), and Digestion (MAPLC3B) between DM and DF group... (D) Volcano plots show DEGs between DFU and DM in blood.(E) Venn plot displays up- and down-overlapping DE-ERGs between DFU and DM. Cut-off criteria: |log2FC| > 0.5, and adj. p < 0.05. (F) Volcano plots show DEGs between DFU and DM in skin. (G) Expression level SIAH2 in DF an DFU group.(H) ROC curve of SIAH based on GSE134431. (I) Expression level ALOX12 in DF an DFU group.(H) ROC curve of ALOX12 based on GSE134431.

Based on the transcriptome sequencing results for patients with DFU and DM, the ssGSEA score for efferocytosis was calculated (Fig 1A), revealing that significantly elevated efferocytosis activity in DFU specimens compared to DM controls. Moreover, the activity of the efferocytosis pathway was estimated and compared between patients with DFU and DM using GSEA, revealing the activation of efferocytosis in patients with DFU (Fig 1B). By analyzing a series of markers across different stages of efferocytosis [33,34], we found that in patients with DFU, ICAM3, RAC1, and MAPLC3B corresponded to the “Find-me,” “Internalization,” and “Digestion” stages, were upregulated in DF tissues (Fig 1C). These findings highlight the upregulation of efferocytosis in DFU compared to that in DM; an overview of efferocytosis is shown in S1 Fig.

### Identification of key efferocytosis-related genes in DFU

To minimize technical variability across datasets, batch effects were first corrected using the R package “sva” (S2 Fig). Subsequently, differential expression analysis of efferocytosis-related genes (ERGs) was performed via the “limma” package, applying a threshold of |log2FC| > 0.5 with adjusted P < 0.05.” Volcano plots displayed the process of differential analysis in blood and skin tissue (Figs 1D and 1F, respectively). Two upregulated ERGs, SIAH2 and ALOX12, were identified through overlapping analysis (Fig 1E). Preliminary validation using the GSE134431 RNA expression dataset revealed elevated SIAH2 levels in DFU patients, with an AUC of 0.875 in the ROC curve (Figs 1G, 1H). However, no statistical difference was observed in ALOX12 expression (Figs 1I, 1J).

### Consensus clustering of patients with DFU using ERGs

**Fig 2:**
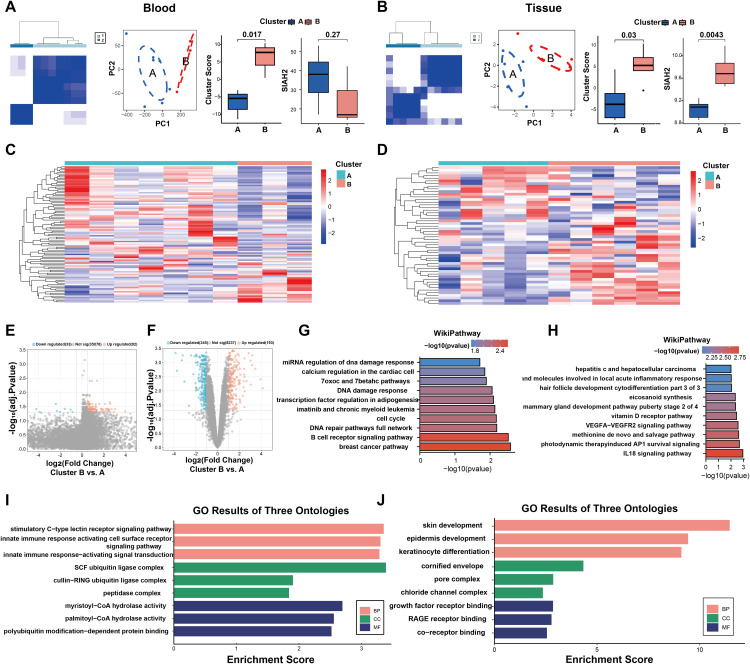
Consensus clustering of patients with DFU based on ERGs. **(A, B)** Consensus matrix, PCA map, and boxplots of cluster scores and SIAH2 expression show the efficiency of clustering in blood and skin tissue.. **(C, D)** Heatmap showing expression of ERGs between DFU and DM, based on data from blood and skin samples. **(E, F)** The volcano plots respectively display the up-regulated and down-regulated differentially expressed genes (DEGs) between different clusters in blood and skin tissues. **(G, H)** The WikiPathway enrichment analysis plot was generated based on DEGs identified from blood and skin tissue samples. **(I, J)** GO enrichment analysis of DEGs in blood and skin, containing BP (Biological Process), CC (Cellular Component), and MF (Molecular Function).

Consensus clustering analysis was conducted on DFU patients utilizing RNA expression profiles derived from both peripheral blood and skin tissue specimens. Based on 100 ERGs, patients with DFU from our sequencing and public cohorts were divided into two clusters. The consensus matrix, PCA reduction plot, and boxplots of cluster scores and SIAH2 expression validated the successful clustering effect (Fig 2A). In the public cohort, the ERG differences were more pronounced between clusters than within clusters (Fig 2B). Heatmaps showing the expression levels of ERGs between clusters A and B in blood and skin tissue (Figs 2C, 2D).

Differential and enrichment analyses were conducted between clusters and explored potential biopathways associated with efferocytosis. DEGs were identified and visualized via volcano plots (Figs 2E, 2G). WikiPathways enrichment analysis highlighted predominant enrichment of efferocytosis-related mechanisms in distinct tissue contexts: B cell receptor signaling pathway and cell cycle regulation in peripheral blood (Fig 2F), alongside IL-18 signaling and VEGFA-VEGFR2 pathways in skin tissue (Fig 2H).GO enrichment analysis further showed the diverse functions of efferocytosis, including participation in the innate immune response and ubiquitin ligase complex in blood (Figs 2I, 2J), and involvement in skin and keratinocyte development and growth factor regulation in skin tissue.

### Expression pattern and functions of SIAH2 in DFU

**Fig 3:**
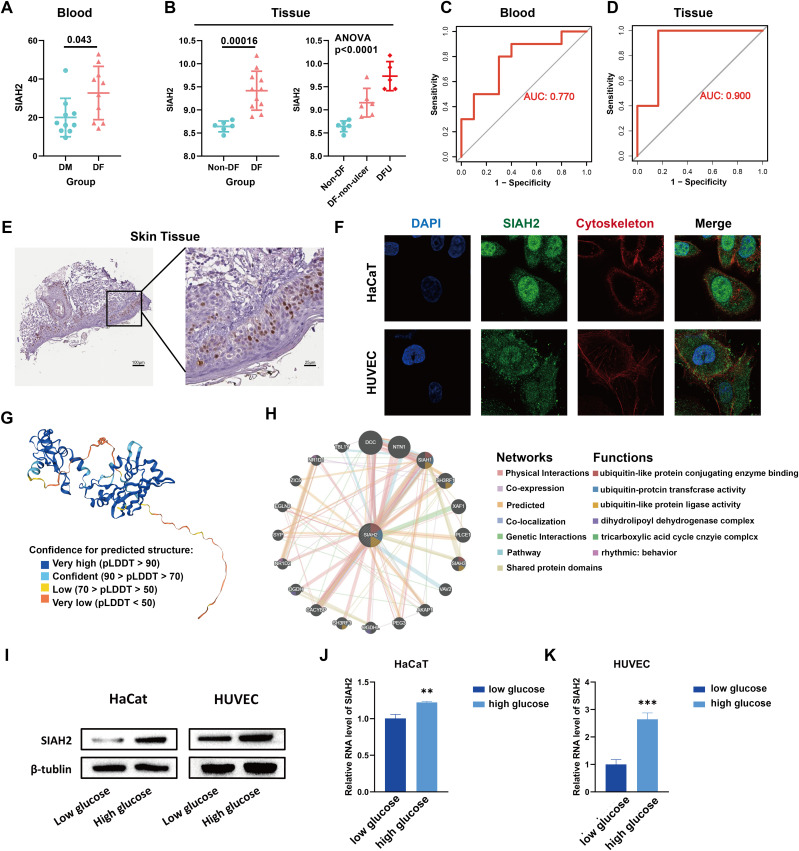
Upregulated expression level and ubiquitination related activity of SIAH2 in DFU. **(A)** The different expression level of SIAH2 in blood between DM and DF patients. **(B)** The SIAH2 expression level in Non-DF, DF without ulcer and DFU patients’ skin tissue. **(C,D)** ROC curves of SIAH2 in blood (AUC = 0.770) and skin tissue (AUC = 0.900) samples; **(E)** Immunohistochemical images demonstrate the expression of SIAH2 in normal skin tissue and its distribution within epithelial cells.; **(F)** The immunofluorescence images illustrate the intracellular expression and subcellular localization of SIAH2 in HUVECs and HaCaT cells.; **(G)** Predicted SIAH2 molecular structure (color indicates confidence); **(H)** PPI image of SIAH2 related genes and function enrichment; **(I)** Western blot images demonstrate the protein levels of SIAH2 in HUVECs and HaCaT cells under low-glucose and high-glucose conditions. **(J, K)** The qRT-PCR demonstrate the RNA levels of SIAH2 in HUVECs and HaCaT cells under low-glucose and high-glucose conditions.

We organized and analyzed the RNA sequencing data from blood and skin samples to gain a comprehensive understanding of SIAH2. SIAH2 was clearly upregulated in DFU compared to the control group, both in the blood and skin tissue. In skin tissue, SIAH2 expression increased progressively from non-DFU skin to DFU skin and DFU, with statistical significance (Figs 3A, 3B). The ROC curve demonstrated the feasibility of SIAH2 as an efferocytosis-related biomarker for DFU, with an AUC of 0.770 in blood and 0.900 in skin (Figs 3C, 3D). Immunohistochemical staining of human skin tissue sections revealed that SIAH2 was primarily expressed in the keratinocyte nucleus (Fig 3E). Immunofluorescence staining of HUVECs and HaCaT cells showed that the SIAH2 content was higher in both the cell nucleus and cytoplasm than in other parts of the cell, which is consistent with the expression of other cell lines in the HPA (Fig 3F,S3 Fig). The molecular structure of SIAH2 is also depicted, with colors representing confidence in the prediction (Fig 3G).

The functions and pathways associated with SIAH2 were also explored. SIAH2 and its related genes were primarily involved in the regulation of the ubiquitination process, tricarboxylic acid cycle, and rhythmic behavior (Fig 3H). Moreover, the function of SIAH2 in the tissues and blood of patients with DFU was analyzed in more detail. We used the gene sets of Hallmark, WikiPathway, and KEGG, and S4 Fig shows the results of GSEA with p < 0.05. Western blot (Fig 3I) and PCR (Figs 3J, 3K) results confirmed that SIAH2 is significantly upregulated in HUVEC and HACAT cell lines in high glucose environments.

### Roles of efferocytosis and SIAH2 in the regulation of the immune microenvironment in DFU

**Fig 4:**
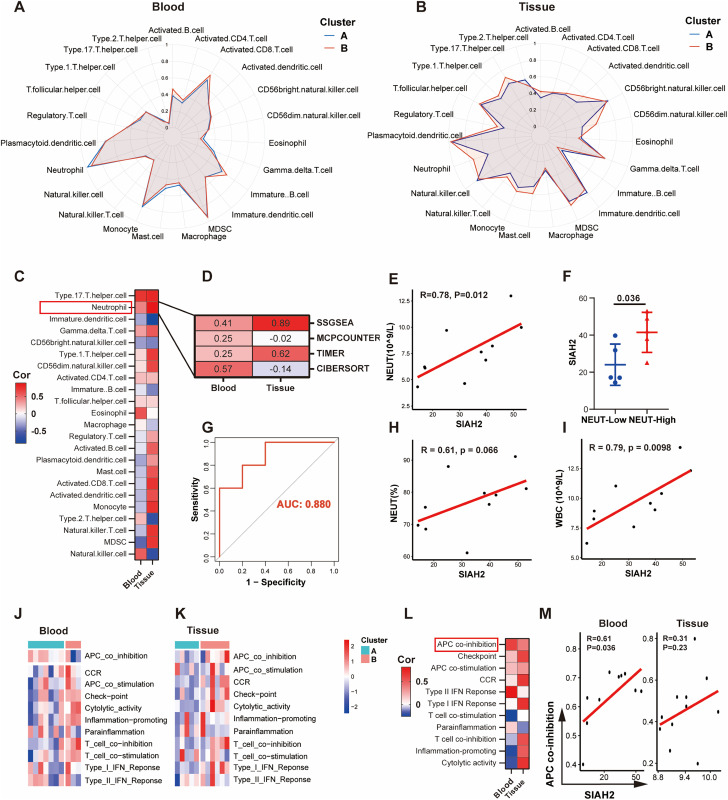
Immune landscape of cluster A and B in DFU based on efferocytosis. **(A, B)** The ssGSEA analysis revealed differences in the infiltration of 23 immune cell types between blood and skin tissues in clusters A and **B. (C)** Heatmap shows correlation between SIAH2 expression and immune cell abundance. **(D)** Neutrophil abundance via ssGSEA, MCPCOUNTER, TIMER, and CIBERSORT in blood and tissue. **(E)** SIAH2 expression correlates with absolute neutrophil count. **(F)** The difference in SIAH2 expression levels among patients with varying abundances of neutrophils in different blood samples. **(G)** ROC curve(AUC = 0.880) indicating the efficiency of SIAH2 expression in predicting the number of neutrophils. (H, **I)** Correlation between SIAH2 expression and “percentage of neutrophils in WBCs” and “absolute number of WBCs”. **(J, K)**The heatmap illustrates the differences in immune function activity between clusters A and B in blood and skin tissues. **(L)** Correlation between immune function and SIAH2 expression level; **(M)** APC co-inhibition is positively correlated with SIAH2 expression in both blood and skin tissue samples.

The previous analyses highlighted the relationship between efferocytosis and immune pathways, prompting an exploration of the immune microenvironment in patients with DFU. Using ssGSEA, the abundance of 23 immune cell types was calculated in blood and tissue samples, revealing differential infiltration of immune cells between clusters (Figs 4A, 4B). The correlation between SIAH2 expression and the relative abundance of immune cells was visualized using a heatmap (Fig 4C). Notably, the relative level of neutrophils in blood exhibited a positive correlation with SIAH2 expression, which was consistent with that in skin tissue. Multi-algorithm analyses further validated the relationship between SIAH2 expression and neutrophil abundance (Fig 4D). In addition, white blood cell (WBC) and neutrophil counts displayed a positive correlation with SIAH2 expression (Figs 4E, 4F). The ROC curve, with an AUC of 0.880, confirmed the accuracy of this correlation (Fig 4G). Moreover, the percentage of neutrophils in WBCs (NEUT%) and the overall WBC count increased with SIAH2 expression (Figs 4H, 4I). Regarding immune function, samples in cluster B exhibited higher activity scores, as observed in the blood and tissue (Figs 4J, 4K). Notably, the activity of APC co-inhibition was positively correlated with SIAH2 expression in both blood and skin tissues (Fig 4L). Further details are provided in Spearman coefficient correlation plots (Fig 4M).

### Analysis of SIAH2 and clinical factors in DFU

**Fig 5:**
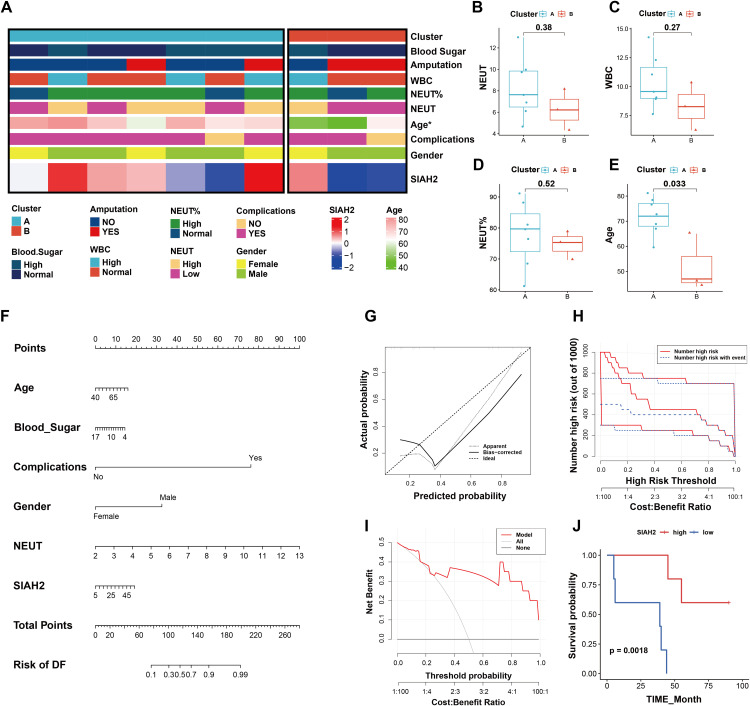
Clinical correlation analysis of DFU patients between cluster A and B. **(A)** Heatmap displaying SIAH2 expression and other clinical factors of DFU patients between cluster A and cluster B; **(B-E)** Boxplots compare NEUT (Neutrophil Count), WBC (White Blood Cell), NEUT% (Neutrophil Percentage) and age between clusters A and B; **(F)** Nomogram model for predicting the occurrence of DFU; **(G)** Calibration curve validates nomogram accuracy. **(H)** Clinical impact analysis evaluates model utility. **(I)** DCA demonstrates model benefits for DFU. **(J)** Kaplan–Meier curve: higher SIAH2 correlates with better prognosis and lower amputation risk (p = 0.0018).

The relationship between SIAH2 expression and clinical factors was explored using the clinical information collected from Qilu Hospital and RNA expression data of patients. The heatmap visualized SIAH2 expression along with several clinical factors related to DFU (Fig 5A). Comparison between clusters revealed a statistically significant age difference only (Figs 5B–5E). A nomogram model was constructed to predict the onset of DFU (Fig 5F), while the calibration plot demonstrated its accuracy (Fig 5G). Clinical impact analysis (Fig 5H) and decision curve analysis (DCA) (Fig 5I) indicated the potential benefits of the model for patients with DFU. In our cohort, higher SIAH2 expression was associated with a better prognosis and a lower risk of amputation in patients with DFU (Fig 5J). Overall, the close relationship between SIAH2 and clinical factors suggests its potential value in clinical practice, which warrants further exploration.

### Overview of the microenvironment of DFU and DM foot tissue at the single-cell level

Based on the single cell sequencing data from GSE165816, we extracted foot skin tissue cells from patients with DM or DFU. These cells were then divided into 29 clusters and re-labeled as one of 14 types of cells (S5A, S5B Figs). The quality of annotation was measured through dot plots of known biomarkers (S5C Fig). Moreover, SIAH2 expression was found in most types of cells (S5D Fig). Interestingly, we found that a sub-group of keratinocytes (Keratinocyte 2) was missing in DFU tissue (S5E Fig). These cells had more significant roles in several metabolism pathways than they did in intercellular junction, cell cycle, and immune pathways (S5F Fig).

### SIAH2 promotes keratinocyte proliferation, migration, and invasion in DFU skin tissue

**Fig 6:**
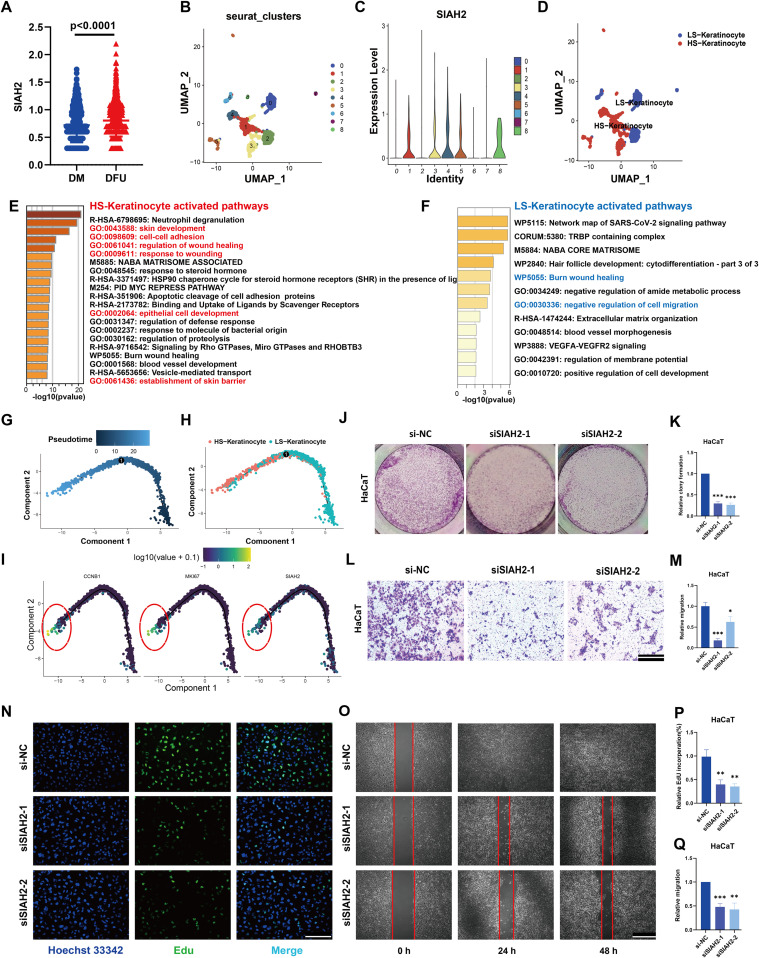
SIAH2 knockdown inhibits keratinocyte proliferation and migration. **(A)** Dot plots comparing SIAH2 expression in keratinocytes between DM and DFU patients; **(B)** Uniform Manifold Approximation and Projection (UMAP) of keratinocytes in DFU patients based on GSE165816 divided into nine clusters. **(C)** SIAH2 expression of the nine clusters. **(D)** Keratinocytes were re-labeled as LS-Ks and HS-Ks by SIAH2 levels.; **(E-F)** Functional enrichment of HS-Ks and LS-Ks. **(G)** Overview trajectory of keratinocytes in DFU patients, color-coded for pseudotime. **(H)**Overview trajectory of LS-Ks and HS-Ks for pseudotime.,(I)The Expression trajectory of CCNB1, MKI67 and SIAH2. DFU: Diabetic foot ulcer, LS-keratinocyte: Low-SIAH2 keratinocyte, HS-keratinocyte: High-SIAH2 keratinocyte; **(J, K)** Representative images and quantitative analysis of the clone formation in HaCaTs with treatment of siNC or siSIAH2. **(L, M)** Representative images and quantitative analysis of the Transwell assays showing the invasive capacity of HaCaTs treated with SIAH2 knockdown. **(N)** The effects of siSIAH2 on the proliferation of HaCaTs were determined via an EdU staining assay; **(O)** Representative images of wound-healing assays demonstrated that migration ability of HaCaTs treated with siSIAH2. **(P)** Quantitative analysis of EdU staining assay. **(Q)** Quantitative analysis of wound-healing assays. The data are shown as the means ± SDs and are representative of three independent experiments. *P < 0.05; **P < 0.01, ***P < 0.001 between the two indicated treatments.

In consideration of keratinocytes as the main component of skin tissue, all keratinocytes were extracted from the single-cell sequencing data. SIAH2 was statistically upregulated in keratinocytes of DFU (Fig 6A). Furthermore, we performed UMAP reduction and divided them into nine clusters (Fig 6B).

According to the SIAH2 expression level in each cluster, they were re-labeled as LS-Ks (low-SIAH2 keratinocyte) and HS-Ks (high-SIAH2 keratinocytes) (Figs 6C, 6D). The highly expressed genes of each keratinocyte subtype were then obtained (cut-off criteria: log2FC > 0.3, and adjusted P < 0.05). We conducted an enrichment analysis of them based on the online tool “metascape” and found that in HS-Ks, functions such as skin development and wound healing were activated, whereas in LS-Ks, cell migration was inhibited (Figs 6E, 6F). Based on the “monocle” package, the differentiation trajectory was plotted and colored by cell subtype and pseudotime, respectively (Figs 6G, 6H). We noticed that SIAH2 expression increased during the differentiation of keratinocytes. Finally, as markers of cell cycle and proliferation, CCNB1 and MKI67 were both highly expressed with SIAH2, indicating their synchronicity of expression, as highlighted (Fig 6I). Plate cloning experiments (Figs 6J, 6K) and EDU proliferation staining (Figs 6N, 6P) showed that the proliferation ability of HaCaT cells was significantly downregulated after SIAH2 was knocked down. Transwell (Figs 6L, 6M) and cell scratch healing experiments (Figs 6O, 6Q) showed that the expression of SIAH2 was positively correlated with keratinocyte migration.

### SIAH2 promotes angiogenesis in DFU skin tissue

**Fig 7:**
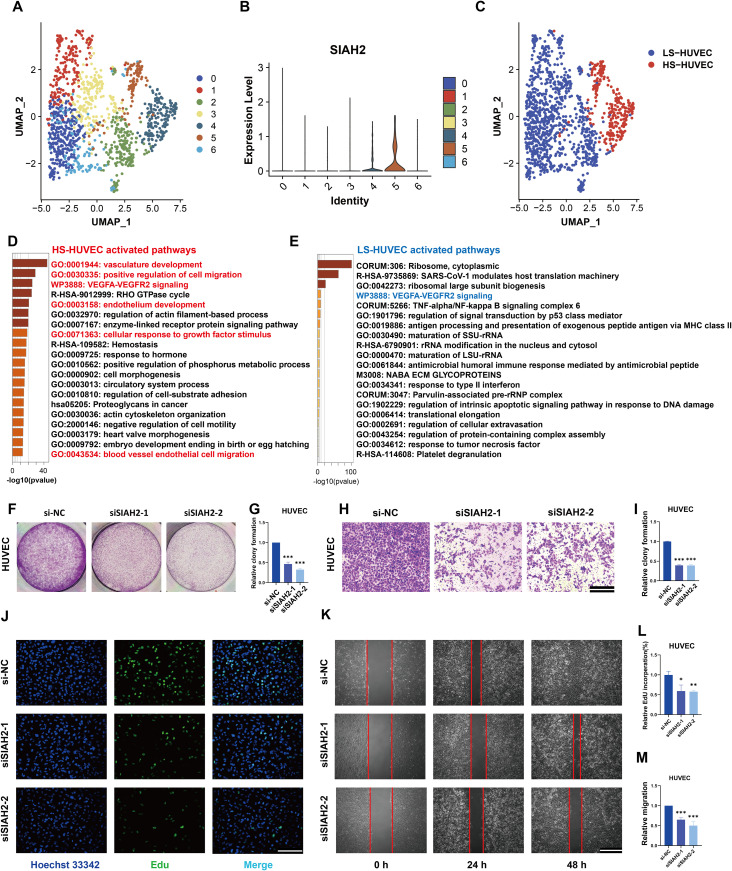
SIAH2 knockdown inhibits endothelial cell proliferation and migration. **(A)** Vascular endothelial cells of patients with DFU from GSE165816 were divided into seven clusters in UMAP; **(B)** SIAH2 expression across clusters; **(C)** cells re-labeled as LS-ECs (blue) and HS-ECs (red) based on SIAH2 levels; **(D–E)** Functional enrichmen of HS-ECs and LS-ECs.; **(F, G)** Representative images and quantitative analysis of the clone formation in HUVECs with treatment of siNC or siSIAH2. **(H, I)** Representative images and quantitative analysis of the Transwell assays showing the invasive capacity of HUVECs treated with SIAH2 knockdown. **(J)** The effects of siSIAH2 on the proliferation of HUVECs were determined via an EdU staining assay; **(K)** Representative images of wound-healing assays demonstrated that migration ability of HUVECs treated with siSIAH2. **(L)** Quantitative analysis of EdU staining assay. **(M)** Quantitative analysis of wound-healing assays. The data are shown as the means ± SDs and are representative of three independent experiments. *P < 0.05; **P < 0.01, ***P < 0.001 between the two indicated treatments.

According to the enrichment analysis, SIAH2 may be related to the regulation of the VEGFA-VEGFR2 signaling pathway, which is an important signal for angiogenesis. To validate this association, we explored the role of SIAH2 in vascular endothelial cells. All vascular endothelial cells were extracted and visualized as shown (Fig 7A). By analyzing these data, we noticed that clusters 4 and 5 had higher SIAH2 expression, and they were then marked as HS-ECs (high-SIAH2 vascular endothelial cells) and LS-ECs (Low-SIAH2 vascular endothelial cells) (Figs 7B, 7C). Their unique highly expressed genes were acquired and enriched (cut-off criteria: log2FC > 0.3, and adjusted P < 0.05). The results showed that HS-ECs had unique functions of vascular endothelial migration and cell proliferation (Fig 7D). In LS-ECs, ribosome-related pathways were activated (Fig 7E). Interestingly, the VEGFA-VEGFR2 signaling pathway was activated in both HS-ECs and LS-ECs, indicating that the role of SIAH2 in angiogenesis did not rely on it. The proliferation, invasion, and migration abilities of HUVECs underwent the same changes as those of keratinocytes (Figs 7F-7M).

In summary, our preliminary findings indicate that SIAH2 expression is closely associated with skin proliferation, repair, and angiogenesis. To further evaluate the specific role of SIAH2 in patients with hyperglycemia and determine whether its upregulation reflects active repair processes or purely pathological manifestations, we performed SIAH2 knockdown in HUVECs and HaCaT cells under different glucose concentrations and conducted Transwell assays (S6 Fig,S7A, 7B Figs). The results suggest that SIAH2 promotes the migratory repair of these cells in both high- and low-glucose environments. Notably, SIAH2 knockdown under high-glucose conditions resulted in more severe impairment of cell migration, indicating that SIAH2 expression in hyperglycemic environments may represent a protective response under stress. These findings confirm the critical role of SIAH2 in patients with DFU.

### SIAH2 affects THP1-derived macrophage-mediated efferocytosis

**Fig 8:**
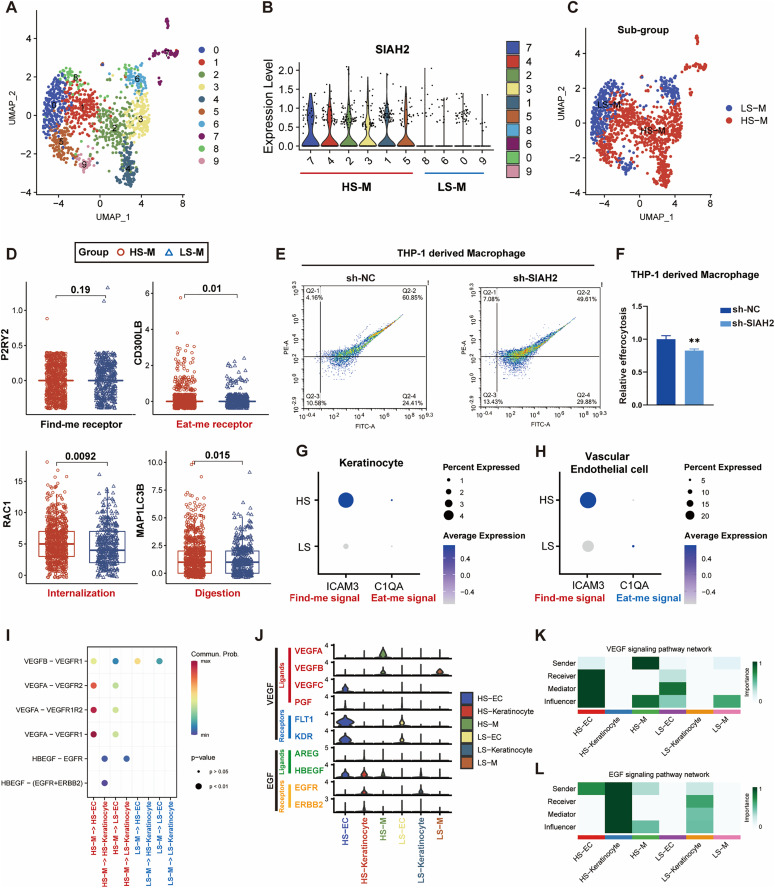
“commuting in both directions” between macrophages and stromal cells mediated by SIAH2. **(A)** UMAP of monocytes/macrophages from GSE165816 showed the cells clustered into 10 groups; **(B)** SIAH2 expression across clusters; **(C)** Re-labeled the monocytes/macrophage as LS-Ms (blue) and HS-Ms (red) by SIAH2 levels; **(D)** Expression of “find me” (P2RY2), “eat me” (CD200LB), internalization (RAC1), and digestion (MAP1LC3B) biomarkers; **(E–F)** Flow cytometry analysis and statistical analysis to detect efferocytosis of macrophages with transfection of shNC and shSIAH2; **(G)** Dot plots of “find me”(ICAM3)/“eat me”(C1QA) signals in HS-Ks release and LS-Ks; **(H)** Dot plots of “find me”/“eat me” signals in HS-ECs and LS-ECs. **(I)** Prediction of communication between mono-cytes/macrophages and stromal cells through VEGF and EGF signals; **(J)** Expression level of VEGF and EGF signals, including receptors and ligands signal; **(K, L)** VEGF and EGF signaling network in DFU tissue’s cells with different expression level of SIAH2.

To preliminarily verify whether SIAH2 affects the prognosis of patients with DFU through efferocytosis-related effects, we first divided monocytes/macrophages into high-SIAH2 monocytes/macrophages (HS-Ms) and low-SIAH2 monocytes/macrophages (LS-Ms) (Figs 8A–8C), and then we compared the expression of efferocytosis biomarkers between them. The results indicated that SIAH2 could promote the reception of the “eat me” signal in macrophages and thus accelerate the digestion of apoptotic cells (Fig 8D). To validate this, we knocked down the expression of SIAH2 in macrophages and found that their engulfment function declined (Figs 8E, 8F). In addition, as apoptotic cells with the potential to be eaten, HS-Ks expressed more “find me” and “eat me” signals than LS-Ks (Fig 8G). Meanwhile, HS-ECs expressed more “find me” signals and fewer “eat me” signals compared with LS-ECs (Fig 8H). In a word, SIAH2 could promote the identification, engulfment, and processing of apoptotic cells in DFU tissue.

### SIAH2 is associated with increased growth factor signaling in DFU tissue

Considering that SIAH2 could promote the proliferation of keratinocytes and endothelial cells and increase the efferocytosis of macrophages to apoptotic cells, the crosstalk between them was unclear. Herein, we deciphered the intercellular communications using “CellChat” and found that in DFU, SIAH2 could promote communication between macrophages and keratinocytes via VEGF signals and relay EGF signals from macrophages to endothelial cells (Fig 8I). In detail, we noticed that in HS-Ms, VEGFA was upregulated, while HBEGF (an EGF ligand) was upregulated in HS-Ms, FLT1 and KDR (VEGF receptors) were upregulated in HS-ECs, and ERBB2 (an EGF receptor) was upregulated in HS-Ks (Fig 8J). Moreover, HS-Ms sent more VEGF signals than LS-Ms, with HS-ECs receiving more signals than LS-ECs (Fig 8K). Similar results were seen in keratinocytes, and interestingly, HS-Ks could send more EGF signals to themselves compared to LS-Ks (Fig 8L). Above all, SIAH2 could promote “commuting in both directions” between macrophages and stromal cells and, in the end, promote the healing of wounds, which is related to increasing levels of efferocytosis.

### In vitro validation of the expression levels of SIAH2 in patients with DFU

**Fig 9:**
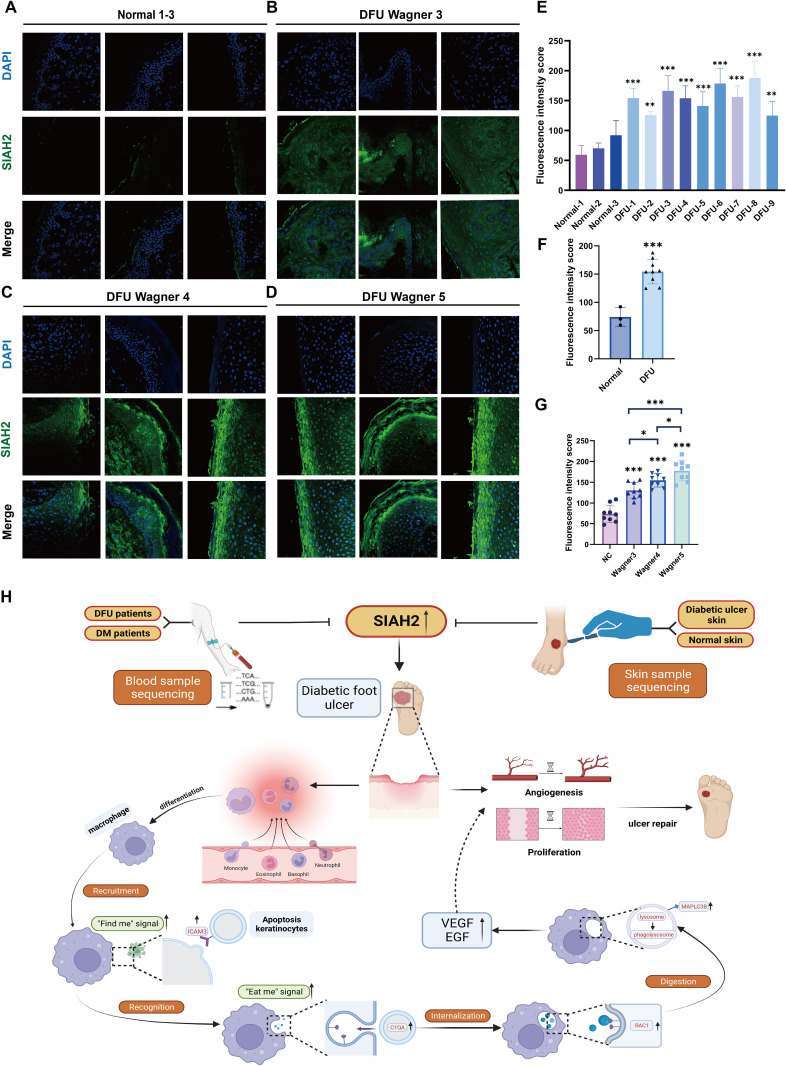
Validation of SIAH2 expression in normal and DFU tissue samples. **(A, B)** Representative images and staining intensity scores of immunofluorescence for SIAH2 (green) in normal (n = 3), DFU Wagner 3 (n = 3), DFU Wagner 4 (n = 3) and DFU Wagner 5 (n = 3) patients. (DAPI stains nuclei blue; scale bar = 100 μm). **(C)** Fluorescence brightness statistics of SIAH2 staining intensity in normal and patients with DFU. **(D)** Quantification of immunofluorescence of SIAH2 staining in different Wagner grades’ patients. **(E)** Flow chart design for this study. The data are shown as the means ± SDs and are representative of three independent experiments. *P < 0.05; **P < 0.01, ***P < 0.001 between the two indicated treatments.

Immunofluorescence results of normal and DFU patients’ skin samples showed that SIAH2 was primarily localized in the keratinocytes of patients with DFU, which is consistent with previous immunohistochemical staining results (Figs 9A–9D). Simultaneously, we evaluated the brightness of fluorescence using ImageJ, and the results confirmed that the fluorescence intensity of SIAH was higher in the skin of patients with DFU than patients without (Figs 9E, 9F). We also found in different Wagner grades, the expression of SIAH2 was upregulated as the grade increased (Fig 9G). The design flowchart of this study is presented in Fig 9H.

## Discussion

Current treatment options for DFU are limited, and there is a need to identify specific therapeutic targets at the molecular and cellular levels to prevent and promote tissue healing in DFU. Previous studies have explored various molecular mechanisms and treatment approaches for DFU, including mesenchymal stem cells and non-coding RNAs [35,36]. Research related to the molecular mechanisms of wound healing in diabetic foot ulcers, especially those related to cell death and clearance of apoptotic cells, such as phagocytosis, autophagy, and efferocytosis, is ongoing [37–39]. Notably, macrophages have been demonstrated to exert pivotal regulatory functions throughout the wound healing cascade. Indeed, several studies have investigated the ability to repair skin ulcers in patients with diabetes from the perspective of combining mesenchymal stem cells with materials such as hydrogel [40,41]. The efferocytosis effect is closely related to the process of apoptosis and the function of phagocytic cells, and is believed to regulate the progression of skin wound healing [42]. Based on above, we conducted a comprehensive identification study on efferocytosis in DFU.

In this study, we performed a comprehensive analysis of blood and tissue gene expression. Patient samples were collected and analyzed in conjunction with the GEO public database to identify changes in various cell death modes and found that efferocytosis was specifically upregulated in patients with DFU.

We then intersected the DEGs with those related to efferocytosis and identified the differential genes closely associated with DFU. As a gene closely related to cell apoptosis and efferocytosis, we have reason to believe that SIAH2 may affect wound healing in patients with DFU by regulating efferocytosis. A previous study suggested that SIAH2 enhances the anti-inflammatory and proliferative abilities of cells and contributes to tumor microenvironment remodeling [43]. Research on the role of SIAH2 has found that it has a regulatory effect on the progression of many tumors of different cell origins [44–46]. This suggests that SIAH2 is closely related to the remodeling of tissues and organs under stress, as well as the proliferation and apoptosis of cells and other life activities.

Our findings revealed that SIAH2 is highly expressed in the blood of patients with diabetes, and its expression in the skin at the ulcer area of patients with DFU is significantly higher than that in normal tissues. Immunohistochemical staining of HPA and clinical samples further confirmed the high expression of SIAH2 in skin keratinocytes. Additionally, the expression of SIAH2 is closely related to the content of white blood cells, particularly the infiltration of neutrophils. This suggests that SIAH2 may regulate acute inflammation in DFU and is also associated with DFU prognosis because of the impact of persistent chronic inflammation on wound healing.

We cultured human immortalized epidermal cells (HaCaT) and human umbilical vein endothelial cells (HUVECs) in different glucose environments and observed a significant increase in SIAH2 expression under high glucose conditions. The reduced dimension identification of single cell groupings showed that there were differences in the expression of SIAH2 in keratinocytes, and the high-expression subgroup was more likely to complete cell proliferation and wound repair. This phenomenon can also be identified in vascular endothelial cells; vascular endothelial cells with high SIAH2 expression tend to complete neovascularization, suggesting that SIAH2 plays a positive regulatory role in the healing process of diabetic foot ulcers, which is consistent with the results of previous clinical studies.

We have proven that the expression of “find me” receptors, such as P2RY2, is relatively stable in phagocytes, while the differential expression of biomarkers such as the “eat me” receptor, internalization biomarkers, and digestion biomarkers is significantly upregulated in the subgroup with high SIAH2 expression. At the same time, the “find me” and “eat me” receptors in endothelial cells are also upregulated, as well as the “find me” receptor in vascular endothelial cells. This suggests that SIAH2 expression has a unique regulatory role in the completion of efferocytosis, indicating that the rise of the overall efferocytosis level corresponding to the fusion statistics of flow cytometry is a macro-level phenomenon derived from changes in multiple independent signals.

Interestingly, cellular communication studies have shown that macrophages with higher expression of SIAH2 emit molecular signals such as EGF and VEGF that promote keratinocyte proliferation and vascular endothelial proliferation. At the same time, the corresponding receptors for these two target cells to receive molecular signals are also significantly increased in the subgroups with high expression of SIAH2. This proves that SIAH2 mediated tissue repair regulation is based on multicellular interaction, which also confirms the importance of SIAH2 in the prognosis of DFU patients.

In summary, this work would be of considerable interest to both the diabetic and broader biomedical communities, as it provides insights into a novel biomarker and predictive model for a condition with significant healthcare implications. However, some aspects of this study require further research. The range of genes related to efferocytosis may not be fixed, and it is likely that additional important efferocytosis regulatory genes have yet to be identified and validated. Moreover, as the number of clinical samples collected in this study is limited, larger specialized studies will be required to validate our findings.

## Conclusion

In this study, SIAH2 was identified as a key gene related to the progression of diabetic foot ulcer (DFU) wound healing. There is an elevated efferocytosis activity in patients with diabetic foot ulcer (DFU) compared to those with diabetes mellitus (DM). SIAH2 was identified as an upregulated gene linked to efferocytosis in DFU, proposing its potential as a clinical biomarker. Protective upregulation of SIAH2 cleaned up apoptotic cells and promoted the release and reception of active biological factors in an efferocytosis dependent approach. The research emphasizes the role of efferocytosis in tissue repair for DFU patients and presents a nomogram model, demonstrating promise for predicting DFU onset and potentially impacting clinical practice. The work would be of considerable interest to both the diabetic and broader biomedical communities, as it provides insights into a novel biomarker and predictive model for a condition with significant healthcare implications.

## Supporting information

S1 Fig Overview of 100 ERGs. (A) Protein-protein interaction network of 100 ERGs. (B) Top-10 hub genes of ERGs and their ranks of degree score. (C) Enrichment analysis network of ERGs. (D) Bar plot showing the diverse functions of efferocytosis.
(PNG)

S2 Fig Batch process of merged datasets. (A, B, C) Boxplots, density curves, and UMAP visualization illustrating the distribution and integration of the merged datasets prior to normalization. (D, E, F) Boxplots, density curves, and UMAP projection demonstrating the same datasets after normalization and batch correction. GSE2114232 was considered as unqualified sample and removed.
(PNG)

S3 Fig SIAH2’s expression in different cell lines. (A, B, C) The immunofluorescence showed the location of SIAH2 in HepG2, U2OS and MCF-7.
(PNG)

S4 Fig GSEA of SIAH2 in blood and skin tissue of patients with DF. (A, B, D) Hallmark, Wikipathway, and KEGG pathways associated with SIAH2 in skin tissue. (C) Enriched KEGG pathways in blood. Red and blue characters representing pathways whose activity positively and negatively correlated with SIAH2 expression respectively.
(PNG)

S5 Fig Overview of microenvironment of DFU and DM foot skin tissue in single cell level. (A) Cells were divided into 29 clusters. (B) Annotation of clustered cells. (C) The quality of annotation was indicated by expression of known markers. (D) Expression of SIAH2 among 13 types of cells. (E) UMAP of cells splitted by tissue type, with cluster “keratinocyte 2” missing in DFU. (F) Unique functions of keratinocyte 1 and 2.
(PNG)

S6 Fig Verification of SIAH2 knockdown efficiency Knockdown efficiency of siRNA. (A, B) Three different sequences verified by PCR in HaCaT and HUVEC cell lines. (C, D) Analysis of the knockdown efficiency of two siRNAs targeting SIAH2 in HaCaT and HUVEC cell lines via Western blotting.
(PNG)

S7 Fig (A, B) Images and statistical results of Transwell assays showing the migration capacity of HaCaT and HUVEC cells treated siSIAH2/siNC cultured with different concentrations of glucose. (PNG)

S1 Table List of efferocytosis-related protein genes. (XLSX)

S2 Table Clinical information of DFU and DM without ulcer patients. (XLSX)

S3 Table SiRNA and primer sequences of SIAH2. (XLS)

S1 Data Original Western Blot images. (PDF)
