# Supplementary figures and images for: Identification of Seven in absentia homolog 2 as a potential efferocytosis-related biomarker in diabetic foot ulcers
Source: PLoS One. 2025 Nov 3;20(11):e0334163. doi: 10.1371/journal.pone.0334163 (PMC12582498; doi:10.1371/journal.pone.0334163)

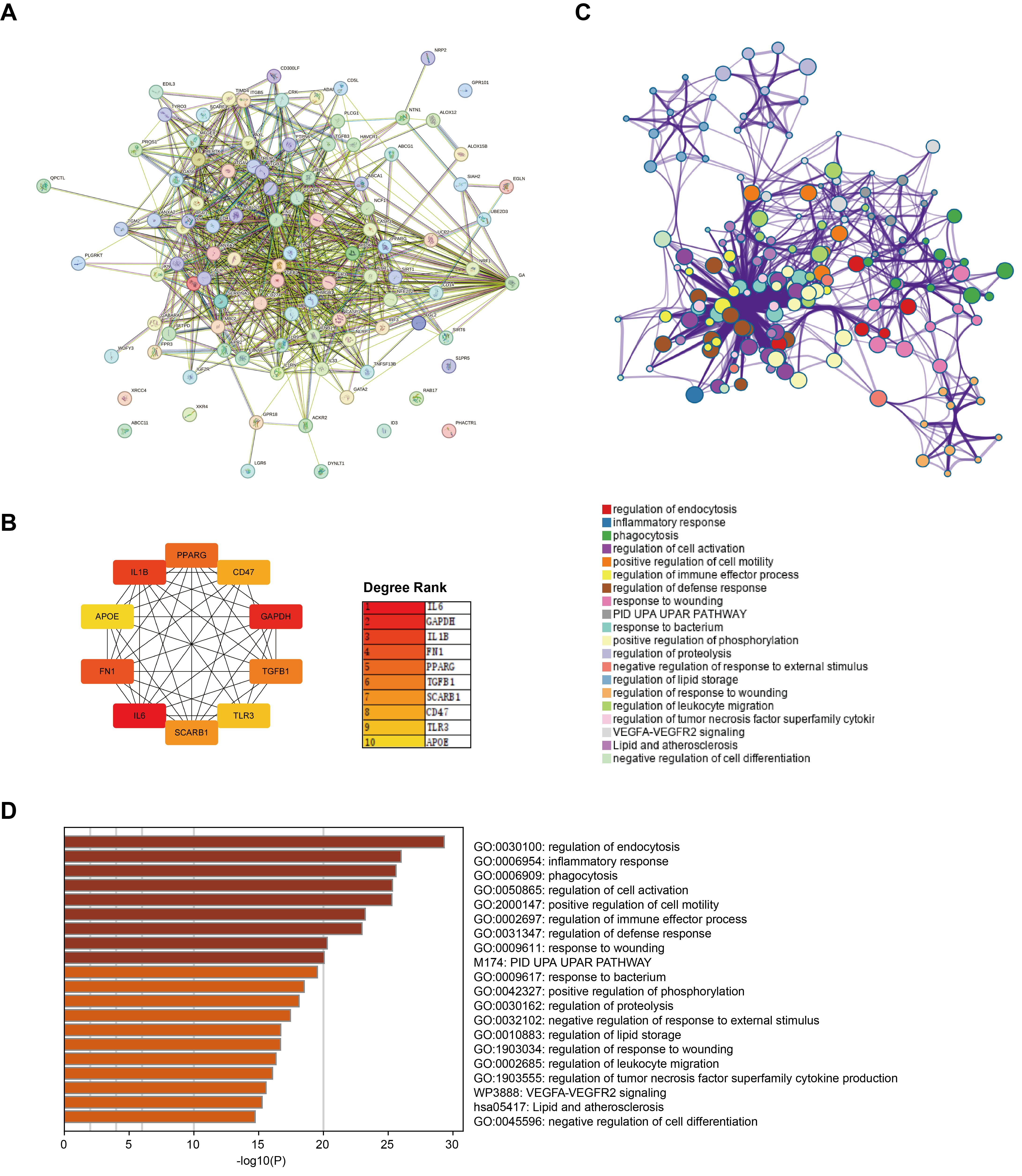

Supplement: S1 Fig — (A) Protein-protein interaction network of 100 ERGs. (B) Top-10 hub genes of ERGs and their ranks of degree score. (C) Enrichment analysis network of ERGs. (D) Bar plot showing the diverse functions of efferocytosis. (PNG) [file pone.0334163.s001.PNG]

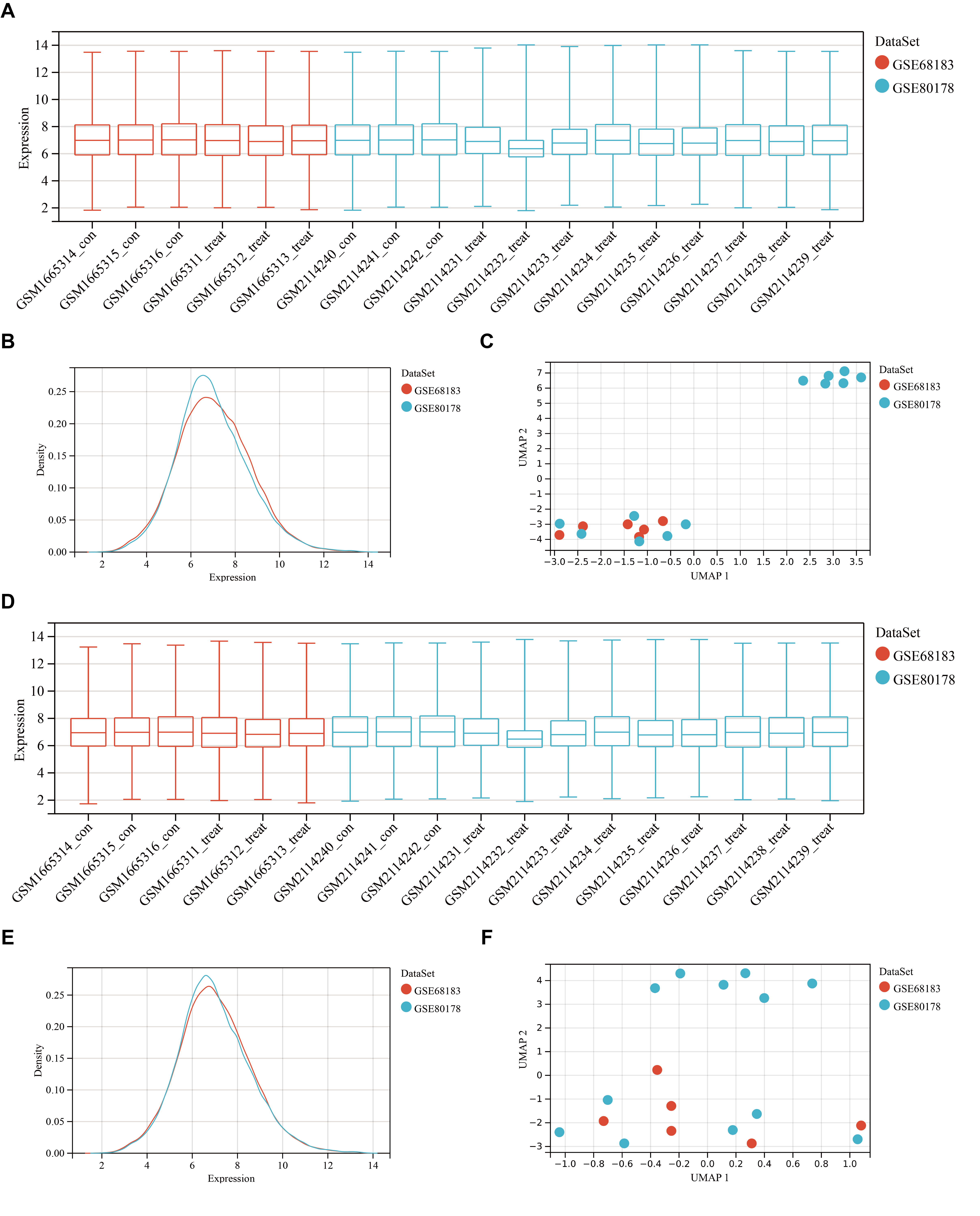

Supplement: S2 Fig — (A, B, C) Boxplots, density curves, and UMAP visualization illustrating the distribution and integration of the merged datasets prior to normalization. (D, E, F) Boxplots, density curves, and UMAP projection demonstrating the same datasets after normalization and batch correction. GSE2114232 was considered as unqualified sample and removed. (PNG) [file pone.0334163.s002.PNG]

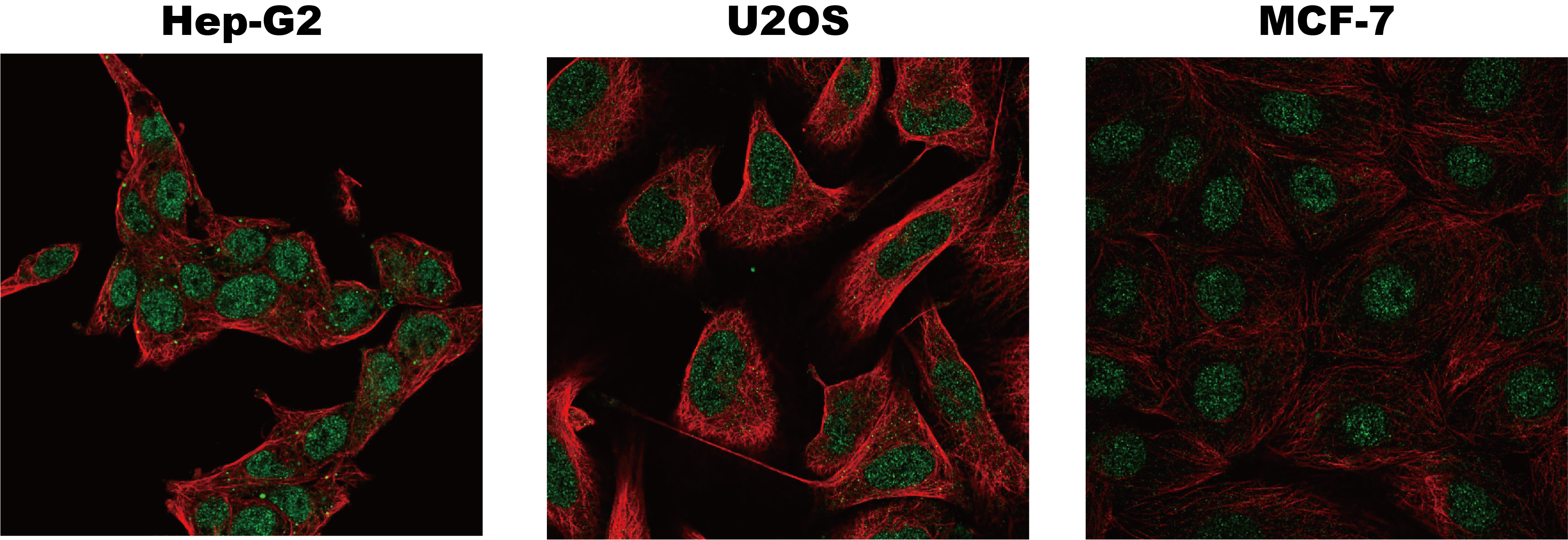

Supplement: S3 Fig — (A, B, C) The immunofluorescence showed the location of SIAH2 in HepG2, U2OS and MCF-7. (PNG) [file pone.0334163.s003.PNG]

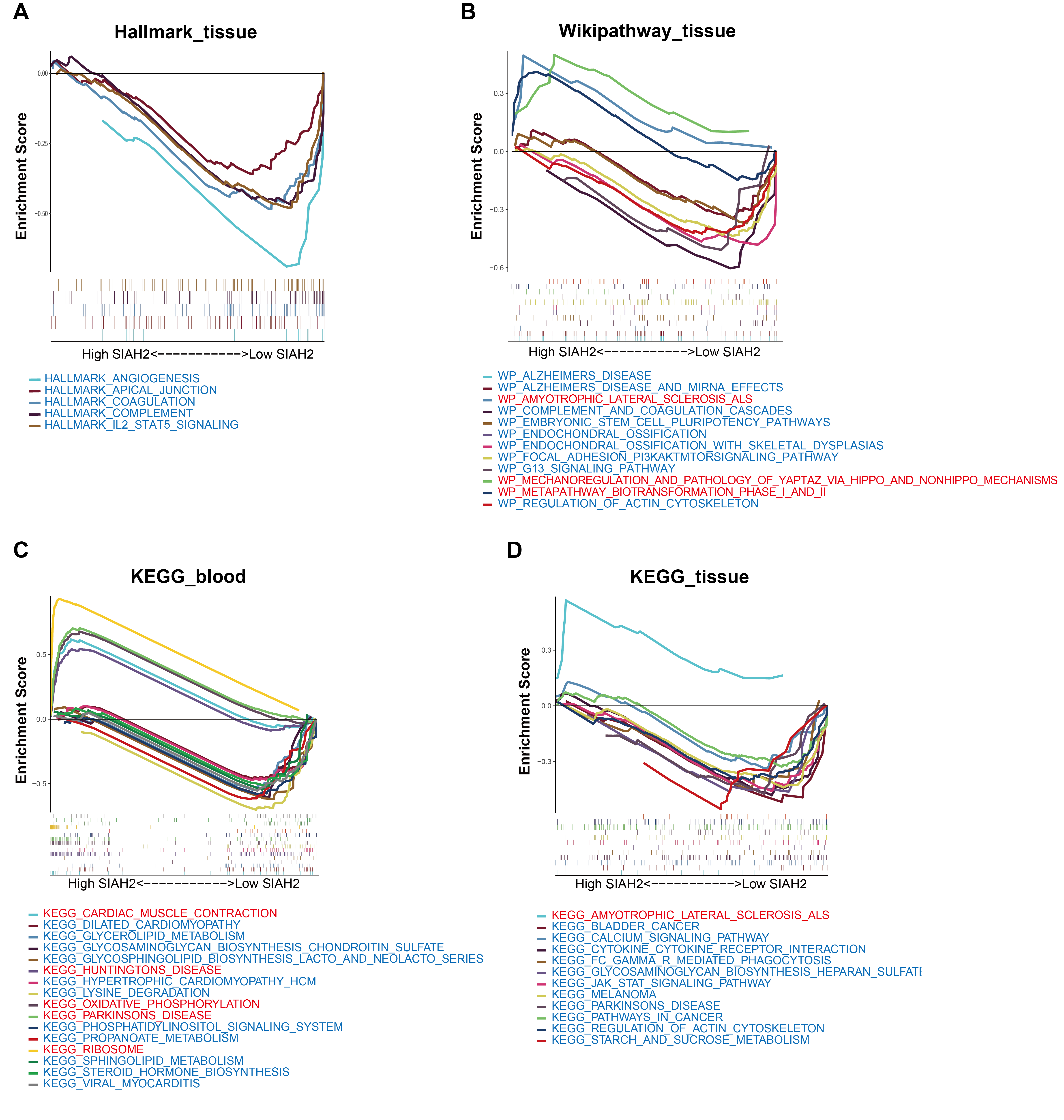

Supplement: S4 Fig — (A, B, D) Hallmark, Wikipathway, and KEGG pathways associated with SIAH2 in skin tissue. (C) Enriched KEGG pathways in blood. Red and blue characters representing pathways whose activity positively and negatively correlated with SIAH2 expression respectively. (PNG) [file pone.0334163.s004.PNG]

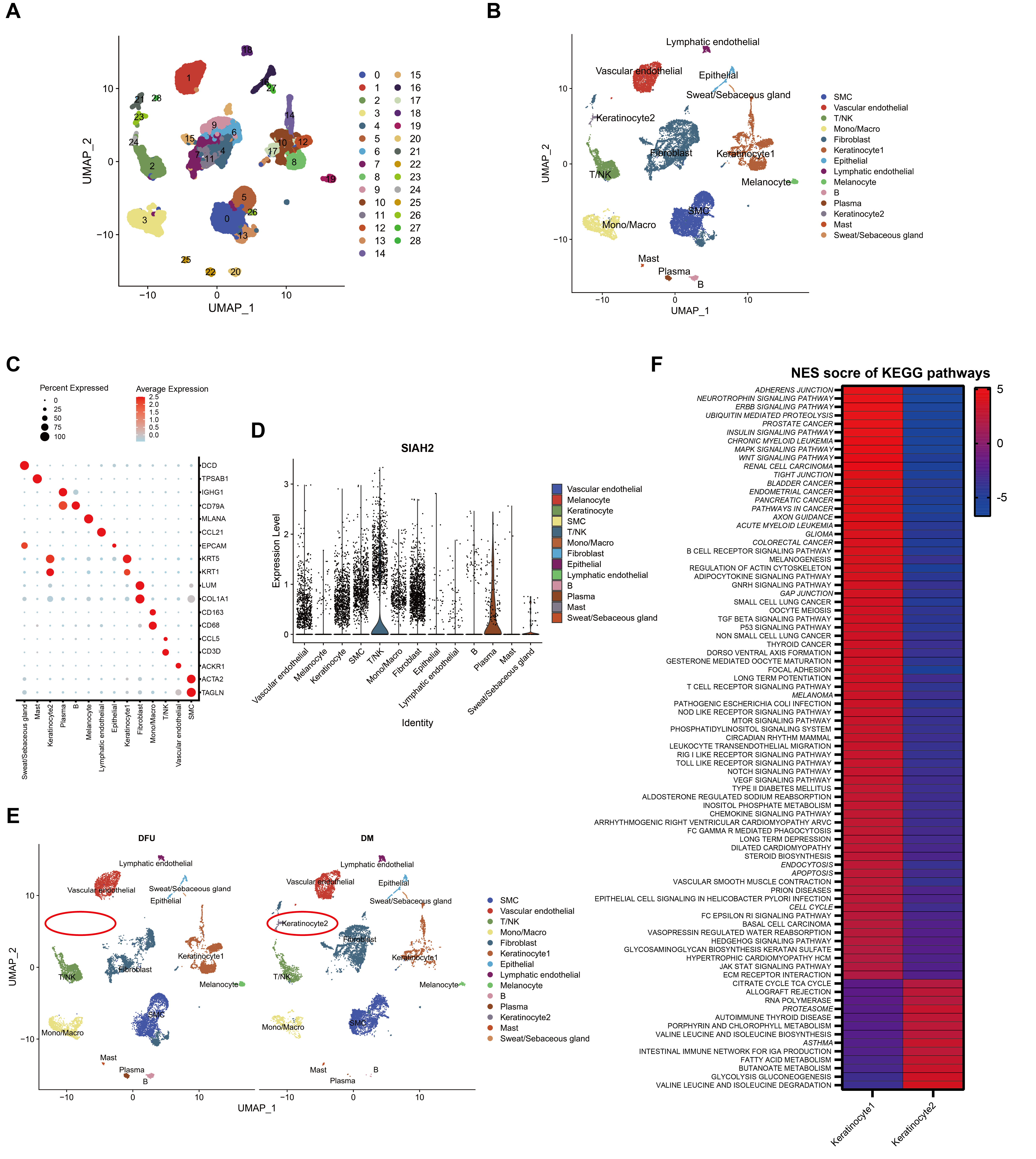

Supplement: S5 Fig — (A) Cells were divided into 29 clusters. (B) Annotation of clustered cells. (C) The quality of annotation was indicated by expression of known markers. (D) Expression of SIAH2 among 13 types of cells. (E) UMAP of cells splitted by tissue type, with cluster “keratinocyte 2” missing in DFU. (F) Unique functions of keratinocyte 1 and 2. (PNG) [file pone.0334163.s005.PNG]

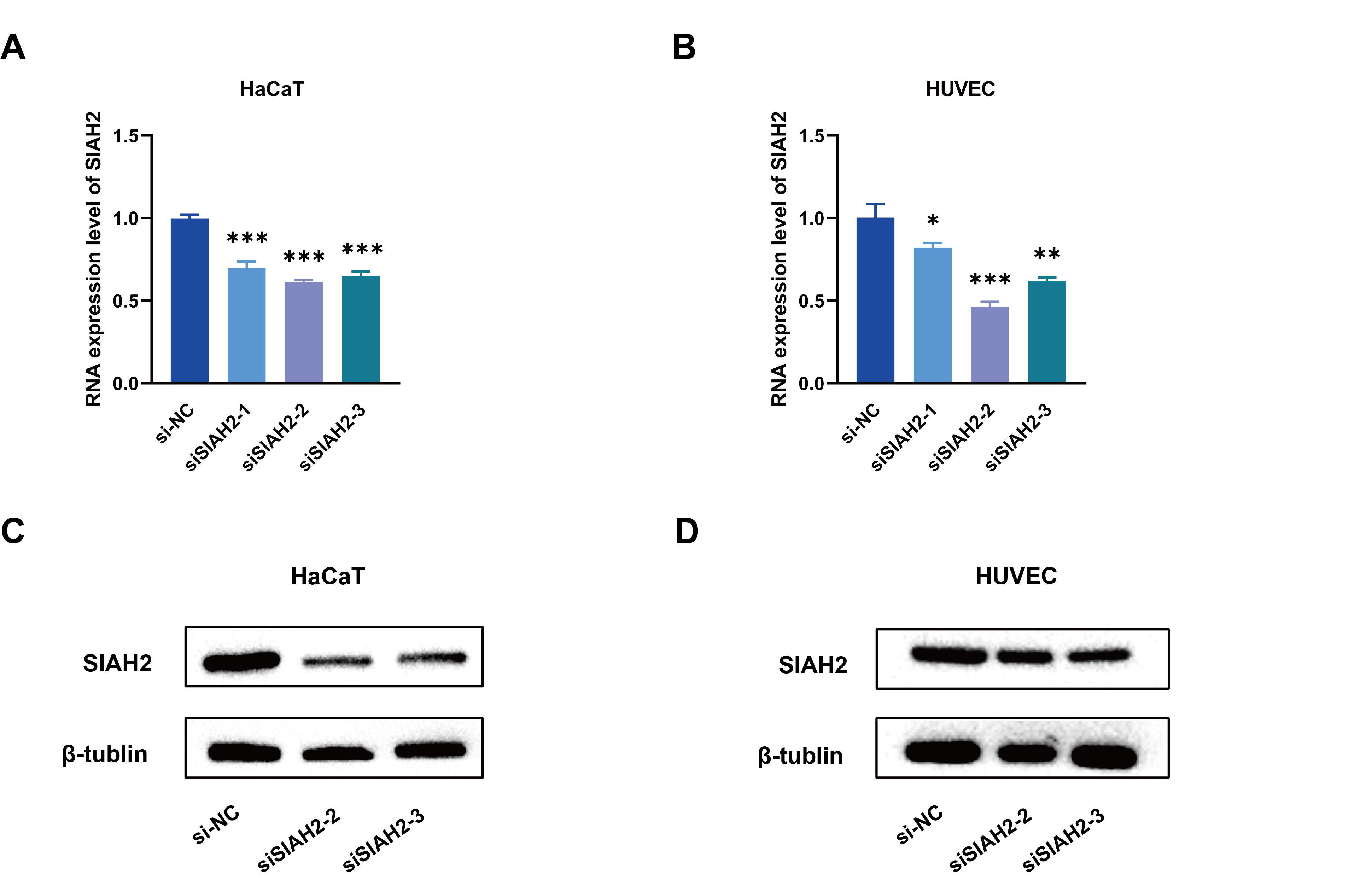

Supplement: S6 Fig — (A, B) Three different sequences verified by PCR in HaCaT and HUVEC cell lines. (C, D) Analysis of the knockdown efficiency of two siRNAs targeting SIAH2 in HaCaT and HUVEC cell lines via Western blotting. (PNG) [file pone.0334163.s006.PNG]

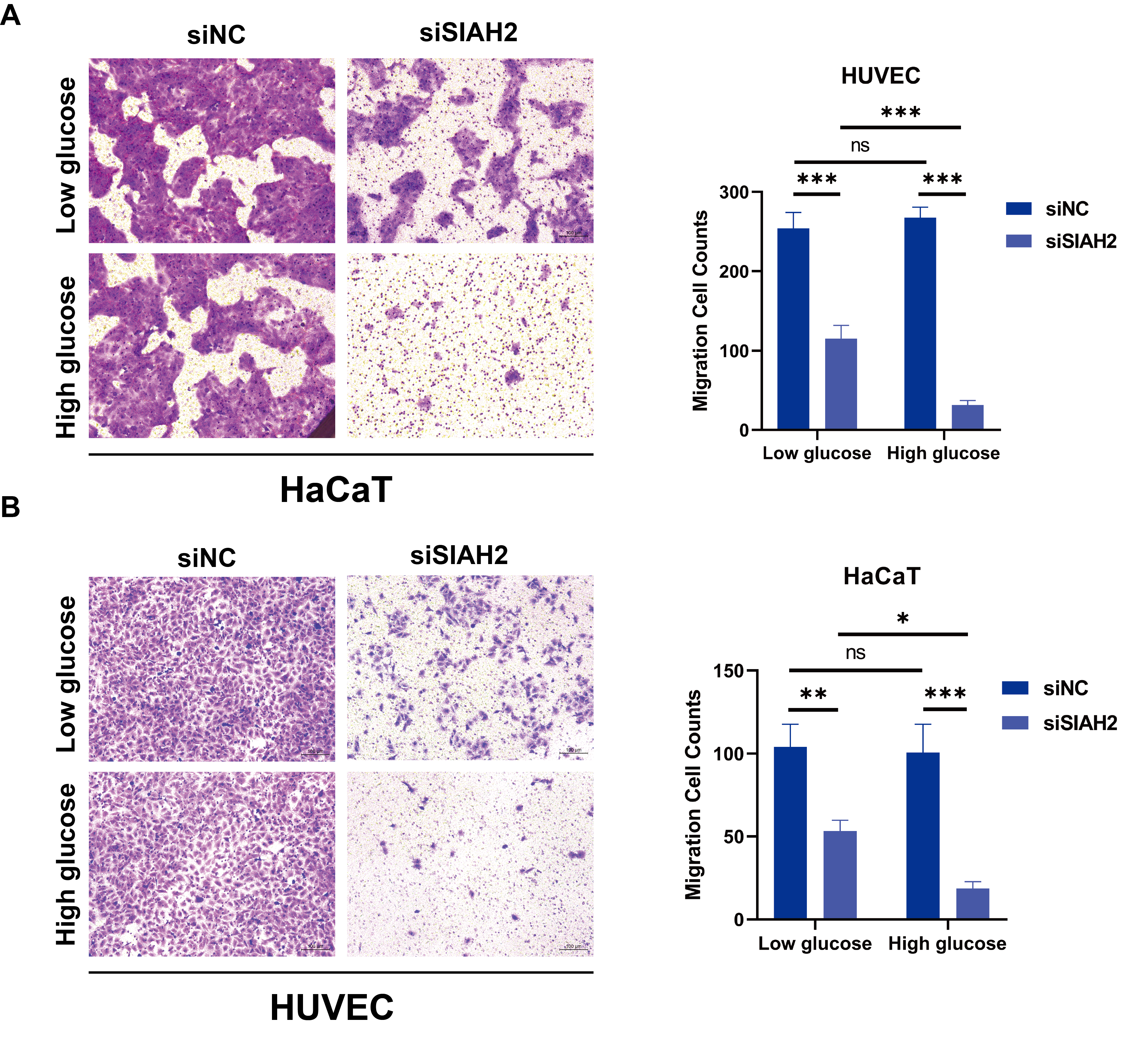

Supplement: S7 Fig — (PNG) [file pone.0334163.s007.png]

Figure 3 I

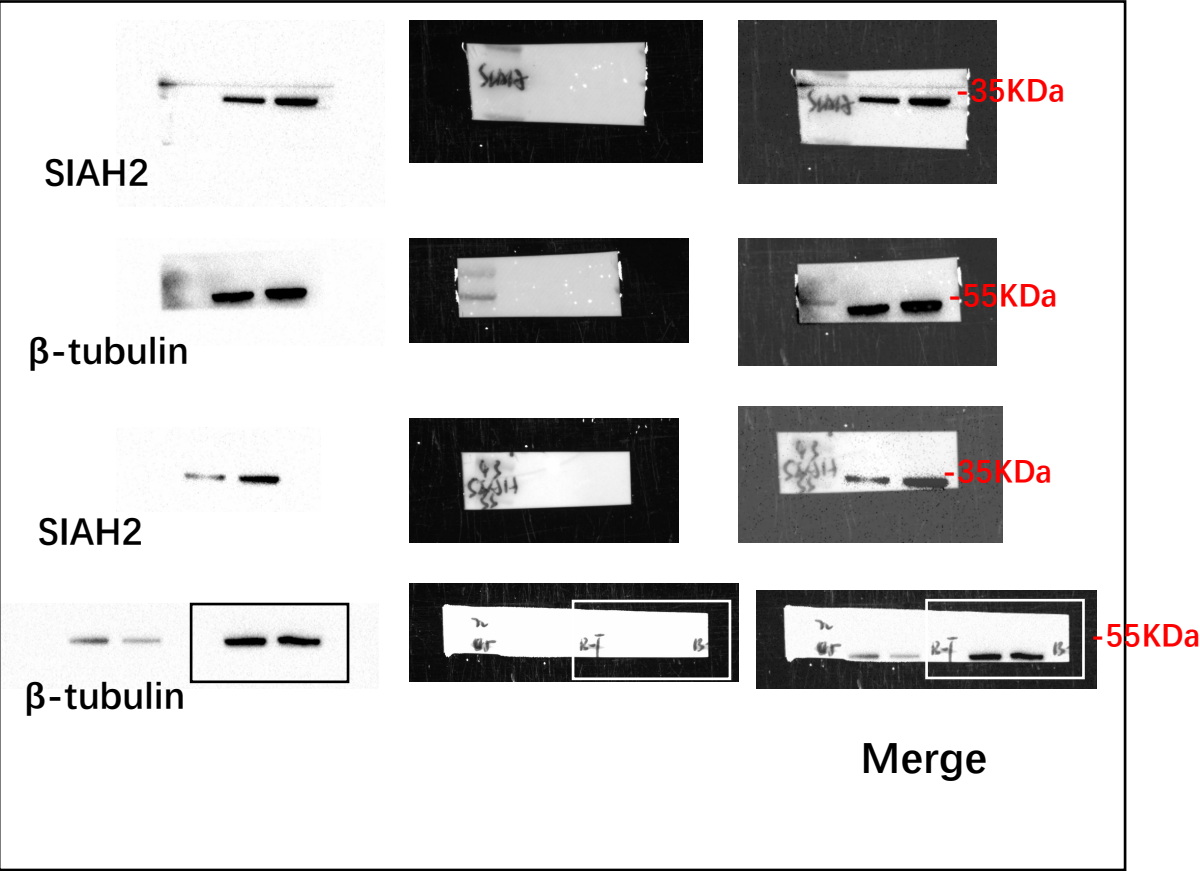

Supplementary Figure 6 C,D

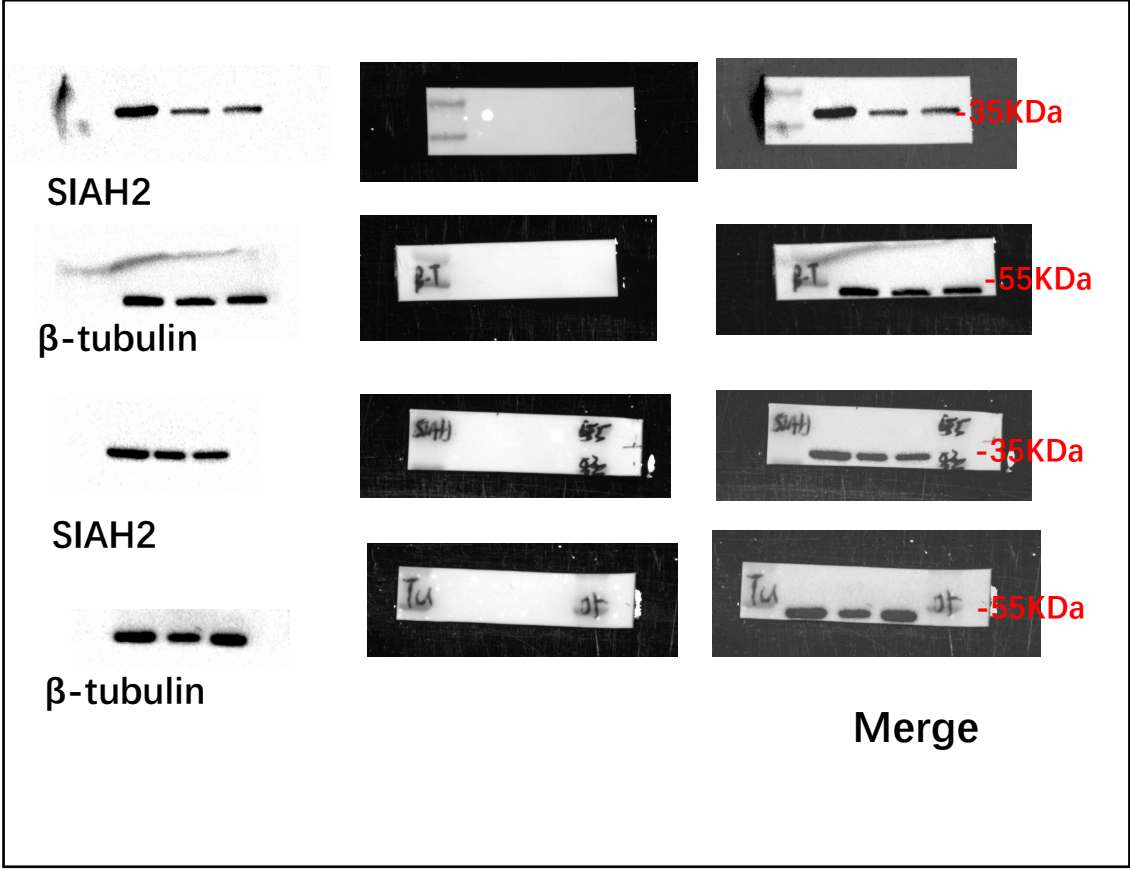

Supplement: S1 Data — (PDF) [file pone.0334163.s011.pdf]
